# Anesthesin. (Para-Amido-Benzoicacidester)

**Published:** 1902-08

**Authors:** Carl Von Noorden

**Affiliations:** Breslau


### TRANSLATION.

### Anesthesin.

(Para-amido-benzoicacidester.)

A LOCAL ANESTHETIC.

By PROF. CARL VON NOORDEN, of Breslau.

(*Ber. klin. Wochensch., April 28, 1902.*)

THE ethyl-ester of P-amido-benzoicacid $C_6H_4<^{NH_2}_{COOC_2H_5}$ was first prepared by Dr. E. Ritsert, of Frankfort on the Main, in 1890, and was recognised by him as a non-toxic local anesthetic. Although this substance aroused great interest among various pharmacologists as a member of a hitherto unknown group of anesthetics, and was especially welcomed by Filehne as a confirmation of his theory of the anesthetic action of the benzoyl group, clinical proof was lacking because of the difficult solubility of the substance in water which thus afforded no prospect of fulfilling the long cherished desire of securing a non-toxic substitute for cocain. Some seven years later meta-amido-p-oxybenzoic-methyl ester (orthoform) was prepared by Einhorn, and despite its insolubility in water was introduced into therapy; whereupon it now became advisable to subject the forgotten amido-benzoicacidester to clinical examination, and all the more so because orthoform had been criticised for its irritating quality and the relative toxicity which proceeded from its phenol-characteristics. It was expected that these unpleasant collateral effects would be much less noticeable in the case of an amido-benzoicacidester, which was designated anesthesin.

Dr. Ritsert gave this preparation to me some two years ago, since which time I have used it uninterruptedly and possess a very extensive experience with the agent.

Anesthesin is a white powder, without odor or taste. When placed upon the tongue a sense of numbness results. The drug is soluble with difficulty in cold water, somewhat more freely in warm water and very freely in alcohol, ether, chloroform, aceton, fats and oils. It may be mixed with fats of all kinds to form ointments without undergoing decomposition.

At my request, Prof. Benz, of Bonn, instituted certain animal experiments in order to test the toxicity of anesthesin. One of these experiments may be cited in this connection. Anesthesin to the amount of 0.6 gm. (10 grs.) was administered to a rabbit in 20 c.cm. of an oily solution, by means of the stomach tube. On the following day the animal was found in good health with urine normal.

The combined experiments which practically resulted negatively, show that in moderate doses anesthesin exerts no deleterious influence upon the animal organism. Given even in colossal doses, the only notable effect is a transient methemoglobinemia. Renal irritation and methemoglobinuria were never observed. The toxic action of anesthesin is therefore seen to resemble to some extent that of phenacetin, to which it is chemically related; and it appears to be strongest when the drug is dissolved in oil.

Prof. Kobert, of Rostock, also tested this drug in regard to its toxicity. His studies show that anesthesin is non-toxic and that it may be used clinically without reservation.

Further research will be required to determine why the anesthetising power on the sensory nerve. We know that if the undissolved powder, finely divided, is applied directly to an exposed nerve-trunk, the electrical excitability of the nerve is not depressed thereby, nor is the sensibility of the area supplied by the nerve in any way modified (Binz). These investigations should be repeated with oily solutions of anesthesin. Thus far no unpleasant collateral effects have been noted in connection with any method of exhibiting anesthesin. There is, therefore, no necessity to cite individual cases, as a general summary will suffice to give the indications for the new remedy as thus far determined.

Anesthesin may be given by the mouth in powder form, 0.3-0.5 gms. (4 to $7\frac{1}{2}$ grs.) two or three times daily for hyperesthesia of the stomach, by which term is understood the painful sensations which follow the ingestion of food including nausea.

Most of these cases could be described as nervous dyspepsia, although some instances of gastric ulcer were present. In this class of cases anesthesin proved to be at least the equal of chloroform-water, chloralhydrate and orthoform. In a few instances it was superior to any of the latter in intensity and duration of action. I would suggest that in cases of this sort the anesthesin should be given from ten to fifteen minutes after the ingestion of food. The highest daily dose given was 2.5 gms. ($37\frac{1}{2}$ grs.). The direct administration per os also includes the use of anesthesin in the form of lozenges, and the like, for cough and dysphagia. The dose in each lozenge, gum-drop, and the like, should vary from 0.02 to 0.04 gm. ($\frac{1}{3}$ to $\frac{2}{3}$ grs.) When the irritation is located in the pharynx or entrance to the larynx, the action of the drug is extremely sedative, resembling that of the so-called "angina-pastilles," which contain antipyrin and cocain, but lasting much longer. The patients, including many with tuberculous laryngitis, preferred the anesthesin lozenges to the pastilles. An emulsion of anesthesin (10 per cent.) in water and gum tragacanth, a 3 per cent. solution in 45 per cent. alcohol and direct insufflation of the powder, were all used in hyperesthesia of the larynx in order to determine the relative efficacy of the new drug with orthoform. Anesthesin was found to possess all the anesthetic properties of orthoform and to be quite free from irritation. The author found that insufflation of the powder produced the best effect, and that in this form anesthesin is the best known anesthetic for the larynx.

In suppository form, 0.2-0.5 (3 to 7½ grs.) anesthesin to 2.0 (30 grs.) cocoa butter, the drug was tested in tenesmus and painful hemorrhoids. For tenesmus it was found inferior to the opiates and belladonna preparations. But for painful hemorrhoids anesthesin gave remarkably good results. Not only were the pain and pruritis favorably affected, but the hemorrhoids were essentially reduced in size. The superiority over orthoform was marked.

Exhibited in the form of soluble bougies, 0.3 (5 grs.) anesthesin in each, the new drug was used in non-specific vesical tenesmus in three females, by introduction into the urethra. In each case the results obtained were highly satisfactory. The treatment was of short duration. Too much should not be claimed for these results, as simple cocoa-butter bougies are sometimes sufficient to relieve this condition. The results obtained from the use of the anesthesin (10 per cent. anesthesin in lanolin) in certain forms of pruritis were brilliant, and more especially in the pruritis vulva which accompanies diabetes. The author has treated over a dozen cases in which the tormenting symptoms with their attendant phenomena of insomnia and nervous excitement were relieved. In cases of this type the strictest diet, the complete disappearance from the urine of glucose and the exhibition of asperin were all without effect. Cocain ointment, even in toxic doses, failed to produce more than a transitory improvement. In all these cases without exception, the anesthesin ointment brought about rapid improvement and when its use was prolonged a permanent cure resulted. Anesthesin was also used in chronic perineal eczema, eczema of the scrotum, and the like, with similarly good results. Decided improvement in the pruritis appeared to influence favorably the disease itself, although it may simply have indicated that a cessation of itching led to the omission of scratching. Anesthesin ointment was found to be very efficacious in recent intertrigo.

Finally it should be mentioned that anesthesin was of value in a certain number of cases of icteric, nephritic and senile pruritis; in certain cases, especially in icterus, the results were strikingly good. It must be admitted that complete failures were recorded.

In brief, anesthesin has an undoubted anesthetic action in many and varied conditions without any known drawbacks. It is especially devoid of irritating properties.

